# *FILIP1*-associated neuromuscular disorder and phenotypic blending due to paternal UPD6

**DOI:** 10.1093/braincomms/fcae330

**Published:** 2024-09-25

**Authors:** Laura M Watts, David J Bunyan, Edoardo Giacopuzzi, Susan Walker, Gabriella Gazdagh, N Simon Thomas, Volker Straub, Anne-Marie Childs, Joan Forsyth, Julie Vogt, Shagufta Khan, Tracey A Willis, Jenny C Taylor, Alistair T Pagnamenta

**Affiliations:** Oxford Biomedical Research Centre, Centre for Human Genetics, University of Oxford, Oxford OX3 7BN, UK; Oxford Centre for Genomic Medicine, Oxford University Hospitals NHS Foundation Trust, Oxford OX3 7HE, UK; Wessex Genomics Laboratory Service, Salisbury District Hospital, Salisbury SP2 8BJ, UK; Human Technopole, Milan 20157, Italy; Genomics England, London E14 5AB, UK; Wessex Clinical Genetics Service, Princess Anne Hospital, Southampton SO16 5YA, UK; Wessex Genomics Laboratory Service, Salisbury District Hospital, Salisbury SP2 8BJ, UK; John Walton Muscular Dystrophy Research Centre, Newcastle University and Newcastle Hospitals NHS Foundation Trust, Newcastle upon Tyne NE1 3BZ, UK; Paediatric Neuromuscular Disease Unit, Leeds Teaching Hospitals Trust, Leeds LS1 3EX, UK; West Midlands Regional Genetics Laboratory, Central and South Genomic Laboratory Hub, Birmingham B15 2TG, UK; West Midlands Regional Genetics Service, Birmingham Women's and Children's Hospital, Birmingham B15 2TG, UK; West Midlands Regional Genetics Service, Birmingham Women's and Children's Hospital, Birmingham B15 2TG, UK; Muscle Team, Robert Jones and Agnes Hunt Orthopaedic Hospital NHS Foundation Trust, Oswestry, Shropshire SY10 7AG, UK; Oxford Biomedical Research Centre, Centre for Human Genetics, University of Oxford, Oxford OX3 7BN, UK; Oxford Biomedical Research Centre, Centre for Human Genetics, University of Oxford, Oxford OX3 7BN, UK; RILD Wellcome Wolfson Centre, University of Exeter Medical School, Exeter EX2 5DW, UK

We read with interest the study of Roos *et al.*^1^ describing a novel syndrome due to disruption of *FILIP1* (filamin A interacting protein 1). Together with the concurrent reports by Schnabel *et al.*^2^ and Al-Kasbi *et al.*,^3^ 10 affected individuals from 7 families have now been described showing combinations of contractures, microcephaly, facial dysmorphism and intellectual disability/developmental delay. Novel disease–gene associations are increasingly limited to ultra-rare conditions and in some cases take several years to replicate. We provide an update on Family A from Roos *et al.*^1^ which includes description of the *FILIP1*-associated phenotype in adulthood. Using data from the 100 000 Genomes Project (100kGP) and the NHS Genomic Medicine Service (GMS), two newly identified families are also described, including one individual where the nonsense *FILIP1* variant was homozygous due to uniparental disomy (UPD) of chromosome 6.

Mapping of recessive traits through the identification of large regions of homozygosity (ROHs) in the genome of affected individuals is a critical tool in human genetics.^4^ Although most often detected in consanguineous families, where they are spread across the genome, ROHs can also occur due to UPD, where the ROH is restricted to a single chromosome. We performed an exploratory ‘differential ROH’ analysis in 11 507 unsolved patients from the 100kGP (v12 release) to detect UPD (Supplementary material). Plotting the difference in size against the ratio between the largest and second largest ROHs (Fig. 1A) identified three outliers. The first two involved full chromosome isodisomy of chromosomes 1 and 4, respectively. These chromosomes have not been linked to known imprinting disorders, and no strong candidate variants on these chromosomes were identified.

**Figure 1:**
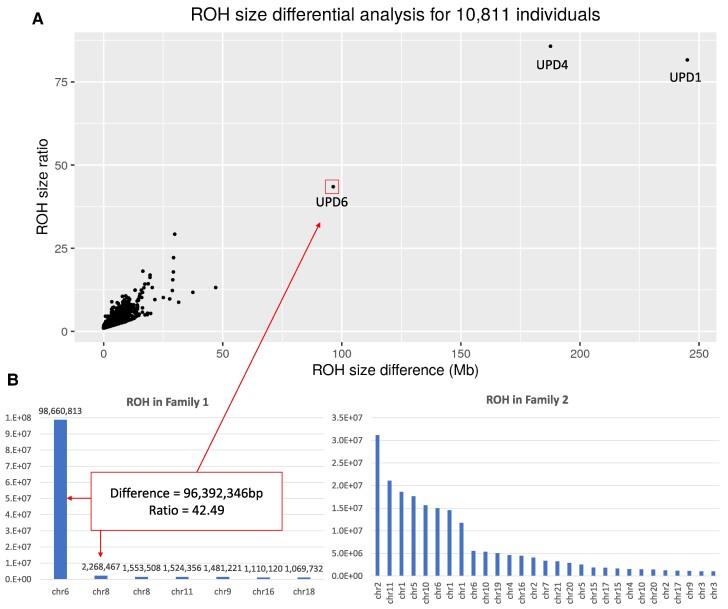
**Differential ROH analysis in our two families identifies biallelic variants in *FILIP1*.** (**A**) Exploratory ROH analysis in patients from the 100k Genomes Project identified three likely cases of UPD involving chromosomes 1, 4, and 6 where a single large ROH region was detected. (**B**) Plots of ROH in descending size order by chromosome in Families 1 and 2. Family 1 has a single large ROH due to UPD6, whereas Family 2 has multiple ROH on different chromosomes, in keeping with consanguinity.

The third outlier (Family 1) was an 11-year-old, born to unrelated white British parents and recruited to the 100kGP under the normalised specific disease ‘arthrogryposis’. Initial analysis using 115 genes on the arthrogryposis panel v2.29 (https://panelapp.genomicsengland.co.uk) and five related panels did not identify any variants of significance. Our differential scan of large ROHs highlighted a 98.7 Mb region on chromosome 6. The next largest ROH was <2.5 Mb in size (Fig. 1B; Supplementary Table 1). Analysis of 210 414 high-quality SNVs confirmed the ROH was due to paternal isodisomy, with heterodisomy across the remainder of this 171 Mb chromosome (Supplementary Figs 1 and 2).

A clinical diagnosis of paternal UPD6 had previously been confirmed using a panel of microsatellites. UPD6pat is linked to intrauterine growth retardation and transient neonatal diabetes mellitus type 1 (MIM #601410). However, additional clinical features included lower limb joint contractures, rocker bottom feet, posteriorly rotated ears, umbilical hernia, shoulder-girdle muscle atrophy, syndactyly, bilateral cryptorchidism, reduced palmar skin folds, neck pterygia, pectus carinatum and a narrow chest (Supplementary Table 2). A paediatric neurology assessment considered that these features were in keeping with reduced foetal movements associated with oligohydramnios or a multiple pterygium syndrome. Muscle biopsy was considered, but not felt to be clinically indicated in the absence of muscle weakness or features indicative of neurogenic or myopathic arthrogryposis. As these features would not be explained by UPD6pat, the family was recruited to the 100kGP. Uniparental disomy can unmask recessive alleles, and so all six rare homozygous variants on chromosome 6 (Supplementary Fig. 3) categorized as TIER3 by the Genomics England pipeline (https://doi.org/10.6084/m9.figshare.4530893.v7) were reviewed. This yielded NM_015687.5(*FILIP1*):c.2152C>T, p.(Arg718Ter) as the most likely causal candidate.

This variant has a CADD score of 37 and is in gnomAD v4.0.0 at a global allele frequency of 142/1,563,902, but never in the homozygous state. No homozygous loss-of-function variants in *FILIP1* are present in gnomAD v4.0.0 or in UK Biobank (https://afb.ukbiobank.ac.uk; accessed March 2024). Of the other five candidates, only NM_000500.9(*CYP21A2*):c.293-4G>A and NM_002526.4(*NT5E*):c.1118T>G involved OMIM-morbid autosomal-recessive genes (www.omim.org). *CYP21A2* is linked to congenital adrenal hyperplasia due to 21-hydroxylase deficiency (MIM #201910), whilst *NT5E* is linked to calcification of joints/arteries (MIM #211800). These conditions did not fit the clinical picture (Fig. 2A–C), and the *CYP21A2* variant is listed in ClinVar as likely benign (VCV000256290.6). Given the new disease association reported recently,^1-3^ c.2152C>T was interpreted as likely pathogenic, based on PVS1_strong, PM2_moderate and PM3_supporting using the ACMG guidelines.^5^ Polymerase chain reaction (PCR)-Sanger validation was performed by the NHS clinical laboratory to confirm this finding (Supplementary Fig. 4).

**Figure 2:**
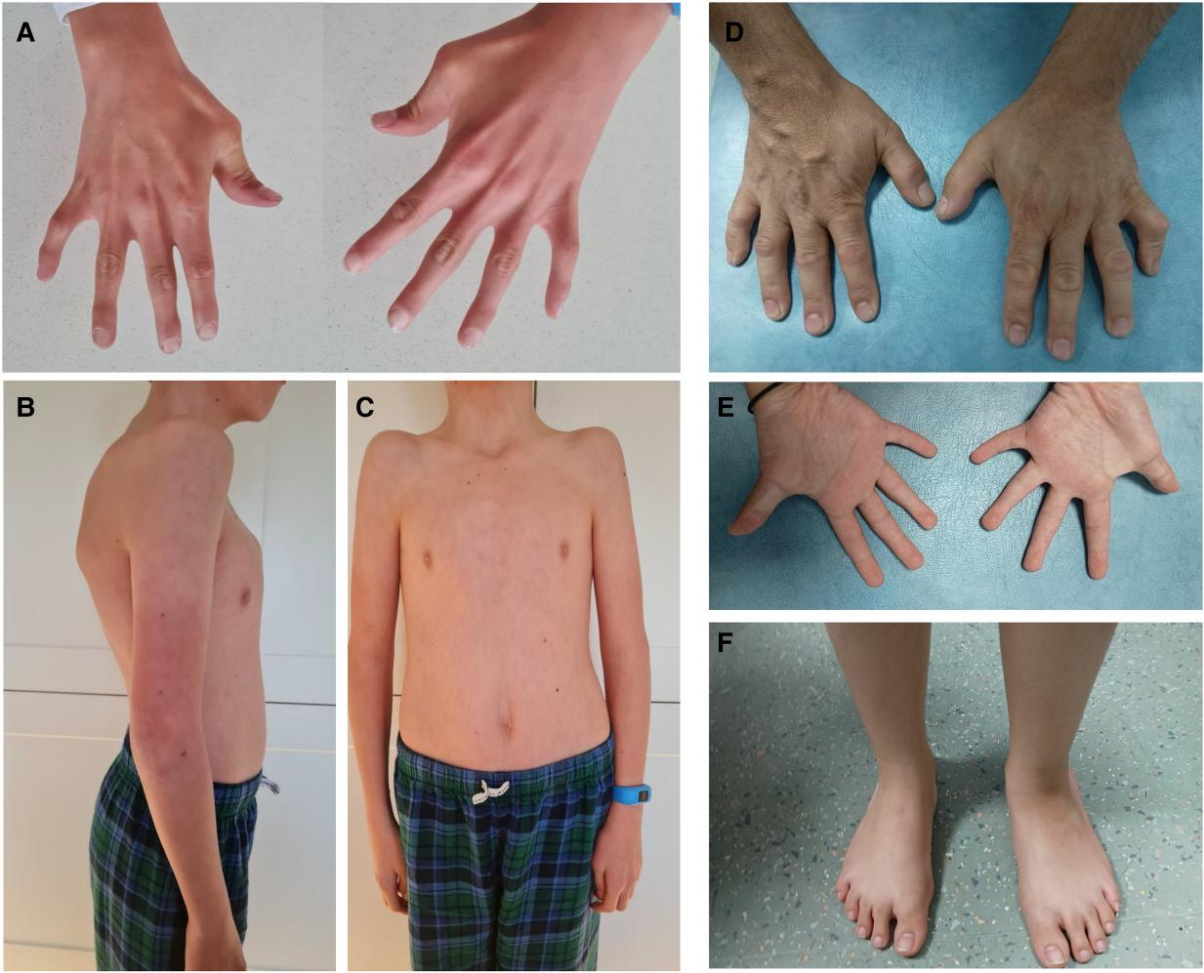
**Photos of individuals from families 1 and 3.** Pictures of the proband from Family 1 show (**A**) webbing of the fingers, camptodactyly and digital contractures, (**B**) pectus carinatum and (**C**) narrow chest with neck webbing. For Family 3, pictures show fifth finger contractures (**D**) for the father and (**E**) for the proband. (**F**) The proband in F3 also has slightly flat feet.

By searching for other families with biallelic truncating variants in *FILIP1*, we identified Family 2, which comprised two sisters recruited to the 100kGP under the indications of congenital myopathy and intellectual disability. Additional clinical features included flexion contractures, scoliosis, facial hypotonia and high arched palate. Both siblings were found to be carriers of a maternally inherited NM_004006.3(*DMD*):c.8713C>T, p.(Arg2905Ter) alteration (Supplementary Fig. 5), not felt to be fully causative of their phenotype. Specifically, the *DMD* variant was inherited from their asymptomatic mother, the older sister showed no evidence of skewed X-inactivation in lymphocytes based upon methylation sensitive analysis of the polymorphic repeat in exon 1 of the human androgen receptor, and there was no abnormality of dystrophin expression on muscle biopsy. Multiple ROHs were identified (Fig. 1B), which included a 15.0 Mb segment spanning *FILIP1,* shared with the affected elder sister. Both sisters were homozygous for a nonsense variant NM_015687.5(*FILIP1*):c.169C>T, p.(Arg57Ter). Through contact with clinicians and analysis of other rare reported variants, it was established that these were the same two siblings as in Family A reported previously.^1^ However, we can now provide follow-up data at ages 27 and 16, allowing the first description of how features of this newly described condition progress into adulthood.

The older sibling (Patient 1 from Roos *et al.*^1^) continues to have proximal muscle weakness and contractures aged 27, although static rather than progressing into adulthood. However, she has developed respiratory failure since the initial assessment reported by Roos *et al.*^1^ Accurate spirometry is limited by restricted mouth opening, but a low forced vital capacity (FVC) worse when supine was noted from adolescence. Overnight pulse oximetry showed some dips in saturations, but was overall satisfactory. More recent deterioration in respiratory function has been noted, and she may be approaching the threshold for non-invasive ventilation. Although there is reported intellectual disability, she works and lives independently. MRI scanning aged 13 demonstrated fatty replacement of gluteus maximus, quadratus femoris, adductor femoris, with slight atrophy of other thigh muscles and sparing of the calves. Repeat scanning aged 22 showed relatively stable appearances, but some asymmetry with the right leg more severely affected than left for reasons which are unclear (Supplementary Fig. 6). Her affected younger sister (Patient 2 reported previously^1^), now aged 16, also has static proximal weakness and contractures. She can hold a pen, but needs help with fastenings and other fine motor tasks. Aged 12, she was reported to have developmental delay and to be 8 years behind her peers. Both siblings have dysarthria, but there has been no report of cardiac problems in either sibling. Of note, the younger sibling has elevated levels of creatine kinase with the suggestion that carrier manifestation for the familial dystrophin variant could be an additional contributing factor. Additional clinical findings are presented in Supplementary Table 2.

Through the NHS GMS, we then identified a third family with a homozygous NM_015687.5:c.463G>T(*FILIP1*), p.(Glu155Ter) variant. This family comprised the proband, with joint contractures, facial dysmorphism and some delayed development, together with her mildly affected father (Fig. 2D–F). The 43-year-old father also carried the same homozygous truncating variant and his phenotype included digital contractures, decreased range of hip and neck movement and mild facial dysmorphism but no reported learning deficit. The proband/father shared ∼40Mb ROHs overlapping *FILIP1*. This is consistent with the multiple consanguineous loops in the pedigree (Supplementary Fig. 7), increasing the chance of disease recurrence in other branches of the family.

Two of the families described here have genetic alterations that suggest phenotypic blending, where the overall patient phenotype is a mixture of two distinct disease phenotypes, a phenomenon previously reported in ∼5% of whole exome sequencing cases.^6^ In Family 1, the transient neonatal diabetes is clinically distinct from the features which can be attributed to *FILIP1*, and the imprinting disorder from UPD6pat was already felt to not explain these additional phenotypic features. In Family 2, both sisters have a maternally inherited *DMD* variant, with a history of walking difficulties and teenage death reported in three maternal uncles. Females carrying *DMD* alterations can manifest features including weakness and learning difficulties, which could overlap with the neuromuscular phenotype of *FILIP1*-associated disorders,^7^ particularly in the younger sibling with elevated creatine kinase levels in the range of those reported in manifesting female *DMD* carriers.^7^

The reports by Schnabel *et al.*^2^ and Roos *et al.*^1^ emphasize different features of the spectrum of this condition. For Family 1 described here, arthrogryposis is the dominant feature, similar to the five individuals described by Schnabel *et al.*^2^ By contrast, Roos *et al.*^1^ report brain malformations, neurodevelopmental delay and myopathy as prominent features in addition to joint contractures. Summarizing the 13 reported cases of the *FILIP1*-associated syndrome, the key features present in almost all are the combination of joint contractures (12/13), motor or speech delay (10/13) and facial dysmorphism (13/13) (Supplementary Table 3). In the 100kGP, there are 163 individuals with arthrogryposis as the recruited condition, and 229 individuals with congenital contractures recorded as an HPO term at recruitment, from which we have only identified three individuals from two families with *FILIP1* alterations, suggesting that this is a rare cause of these features. However, there are only four individuals in 100kGP with the combination of congenital myopathy and ID, two of whom are the individuals reported by Roos *et al.*^1^ and here.

Two of our reported individuals (Patient 1 from Roos *et al.*^1^ and the father of the proband in Family 3) are adults, the first time the adult phenotype of this condition has been reported. Importantly, the contractures appear static rather than progressive, but one individual has developed the serious complication of respiratory failure. Both live and work independently as adults and the diagnosis was only made in the father from Family 3 following investigation of his more severely affected daughter. Neither has been reported to have cardiac complications which can complicate some neuromuscular conditions. The patients described here demonstrate variable disease severity, both between and within families (Family 3). The reasons for this are unclear, but likely represent the combined effects of unidentified genetic modifiers and environmental factors. Further longer-term follow-up is needed to assess whether respiratory failure or other complications become apparent in other affected individuals with age.

## Supplementary Material

fcae330_Supplementary_Data

## Data Availability

100kGP and GMS data are available in the National Genomic Research Library (https://doi.org/10.6084/m9.figshare.4530893.v7).
