# Supplementary material for: FILIP1-associated neuromuscular disorder and phenotypic blending due to paternal UPD6
Source: Brain Commun. 2024 Sep 25;6(5):fcae330. doi: 10.1093/braincomms/fcae330 (PMC11462438; doi:10.1093/braincomms/fcae330)
Supplement: fcae330_Supplementary_Data [file fcae330_supplementary_data.zip › Supplementary material.pdf]

SUPPLEMENTARY MATERIAL: *FILIP1*-associated neuromuscular disorder and phenotypic blending due to paternal UPD6

Supplementary background and methods

Mapping of recessive traits through the identification of large regions of homozygosity (ROHs) shared by multiple affected individuals, has proved to be a critical tool in human genetics.[1] Even in known disease genes, pathogenic variants are overrepresented in large and presence in one of the top 10 such regions can be used as evidence supporting pathogenicity.[2]

Although large ROHs are most often detected in consanguineous populations, ROHs can also occur due to uniparental disomy (UPD). Several different biological mechanisms can lead to UPD.[3] One such mechanism is monosomy rescue, which results in whole chromosome isodisomy (i.e. a single ROH region spanning from telomere to telomere). Other types of UPD can result in mixture of isodisomy and heterodisomy and thus can resemble the ROH regions seen in consanguineous families. However, whilst ROHs due to consanguinity are typically spread across the genome, ROHs due to UPD are restricted to a single chromosome.

In this study, we aimed to identify instances of UPD in 100kGP participants and ultimately find cases with imprinting disorders and potentially novel recessive conditions. ROH data were assessed for 11,507 probands from the 100k Genomes Project (100kGP, data release v12, 6th May 2021). These individuals had been selected due to the absence of a diagnosis in the 100kGP exit questionnaire or Tier 1/2 variants. ROH regions were called by ROHcaller (Illumina) from whole genome single nucleotide variant (SNV) data and were filtered using custom scripts and the dplyr package in RStudio. Cases where the second largest ROH (on a different chromosome to the largest ROH) was >5Mb were removed due to likely consanguinity, leaving 10,811 cases. This ROH threshold was chosen as ROH regions of this size are rare in demonstrably outbred individuals.[4] Plotting the difference in size against the ratio between the largest and second largest ROHs identified 3 outliers (Figure 1A).

Additional biallelic truncating variants in 100kGP participants were identified from the National Genomic Research Library using the secure research environment and the LabKey application. This reports small variants that were called by Platypus[5] and prioritised by Genomics England's TIERING pipeline.[6] Further information about Genomic England's Research Environment and technical documentation relating to the 100kGP are available at https://re-docs.genomicsengland.co.uk/further_reading/. This includes a "Rare Disease Genome Analysis Guide" which includes how potential instances of UPD are flagged by the standard pipeline.

Supplementary Table 1: Regions of homozygosity of >1Mb detected in the respective probands. Genomic coordinates are based on GRCh38. †Large homozygous region restricted to a single chromosome indicative of uniparental isodisomy. In Family 2 the ROH in the proband (younger sister) totalled ~1/18 of the genome, in keeping with the known consanguinity. Shading indicates ROH regions harbouring *FILIP1*. For Family 3, ROH data is only available for chr6 where segments of ~40Mb were seen in both proband (chr6:36,684,112-79,860,168) and father (chr6: 47,735,523-85,068,104).

Chromosome	Start	End	Size
**Family 1**			
chr6	1,008,132	99,668,945	98,660,813†
chr8	133,678,327	135,946,794	2,268,467
chr8	88,072,463	89,625,971	1,553,508
chr11	47,385,041	48,909,397	1,524,356
chr9	110,152,070	111,633,291	1,481,221
chr16	68,397,853	69,507,973	1,110,120
chr18	41,835,496	42,905,228	1,069,732
**Family 2**			
chr2	129,917,442	161,048,311	31,130,869
chr11	211,644	21,272,839	21,061,195
chr1	18,361,115	36,964,729	18,603,614
chr5	13,634,042	31,296,425	17,662,383
chr10	50,350,989	66,007,659	15,656,670
chr6	64,461,526	79,426,859	14,965,333
chr1	39,084,538	53,607,085	14,522,547
chr1	237,058,372	248,832,774	11,774,402
chr6	158,039,574	163,586,390	5,546,816
chr10	108,699,839	114,067,521	5,367,682
chr19	10,062,273	15,119,842	5,057,569
chr4	183,419,806	188,007,657	4,587,851
chr16	85,609,757	90,110,179	4,500,422
chr2	49,332,579	53,410,607	4,078,028
chr7	150,162,009	153,545,485	3,383,476
chr21	13,140,394	16,397,606	3,257,212
chr20	2,333,260	5,233,822	2,900,562
chr5	69,133,099	71,673,087	2,539,988
chr15	73,955,923	75,844,688	1,888,765
chr17	50,284,825	52,125,627	1,840,802
chr15	88,371,403	90,023,402	1,651,999
chr4	181,318,301	182,840,529	1,522,228
chr10	76,238,685	77,709,183	1,470,498
chr20	52,773,084	54,206,102	1,433,018
chr2	100,926,429	102,158,349	1,231,920
chr17	67,404,138	68,580,348	1,176,210
chr9	79,975,821	81,083,219	1,107,398
chr3	99,149,867	100,192,917	1,043,050
chr3	94,957,169	95,988,292	1,031,123

Supplementary Table 2: *Available as separate xlsx file*.

Supplementary Table 3: Summary of reported clinical features of the *FILIP1*-associated syndrome. Numbers represent the incidence of features in the 3 families described here in combination with previously reported families in the literature. Note that not all features were reported upon in previous papers. The sisters reported here as Family 2 and as Family A in Roos *et al*[7] are only counted once. Similarly, the family reported by Al-Kasbi *et al*[8] and reported again in Schnabel *et al*[9] are only counted once.

Feature	Number of cases
Consanguinity	13/13
Reduced fetal movements	3/13
Neonatal hypotonia	6/13
Talipes	7/13
Contractures	12/13
Reduced palmar/plantar skin creases	7/13
Short neck with webbing	9/13
Scoliosis	5/13
Motor delay	10/13
Speech delay	8/13
Intellectual disability	5/13
Microcephaly	5/13
Facial dysmorphism	13/13

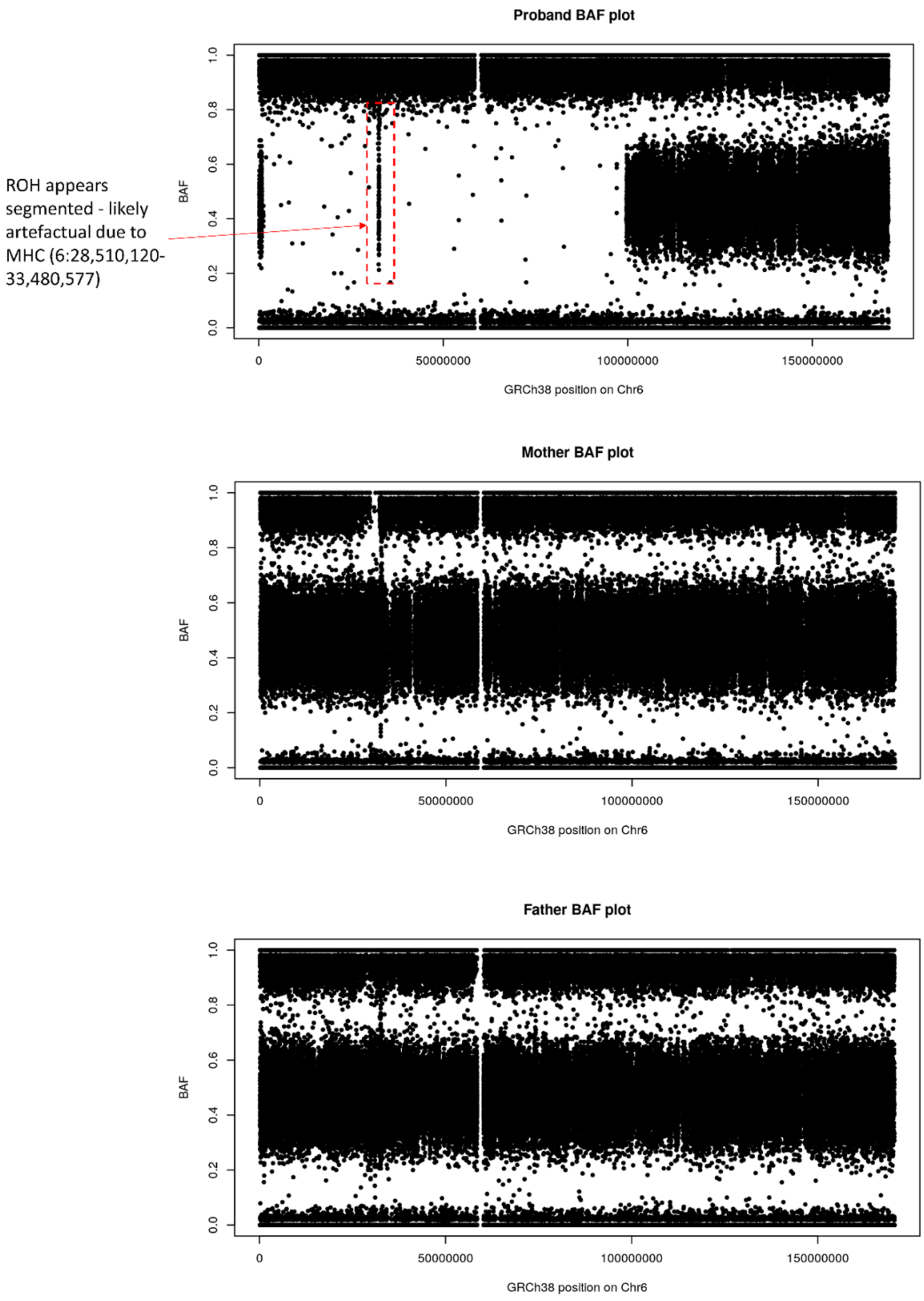

Supplementary Figure 1: B-allele frequency (BAF) plots for chromosome 6 highlight a single 98.7Mb ROH consistent with the algorithmic call. Data was plotted in RStudio just for high-quality SNVs, which were extracted from the multisample platypus vcf file to include only PASS variants with 30-60x coverage in all 3 family members. Indels and variants called as part of phase blocks (i.e. with other

variants nearby) were also removed. The apparent segmentation of the ROH is likely an artefact due to the MHC locus. BAF data for both parents show no significant ROH regions.

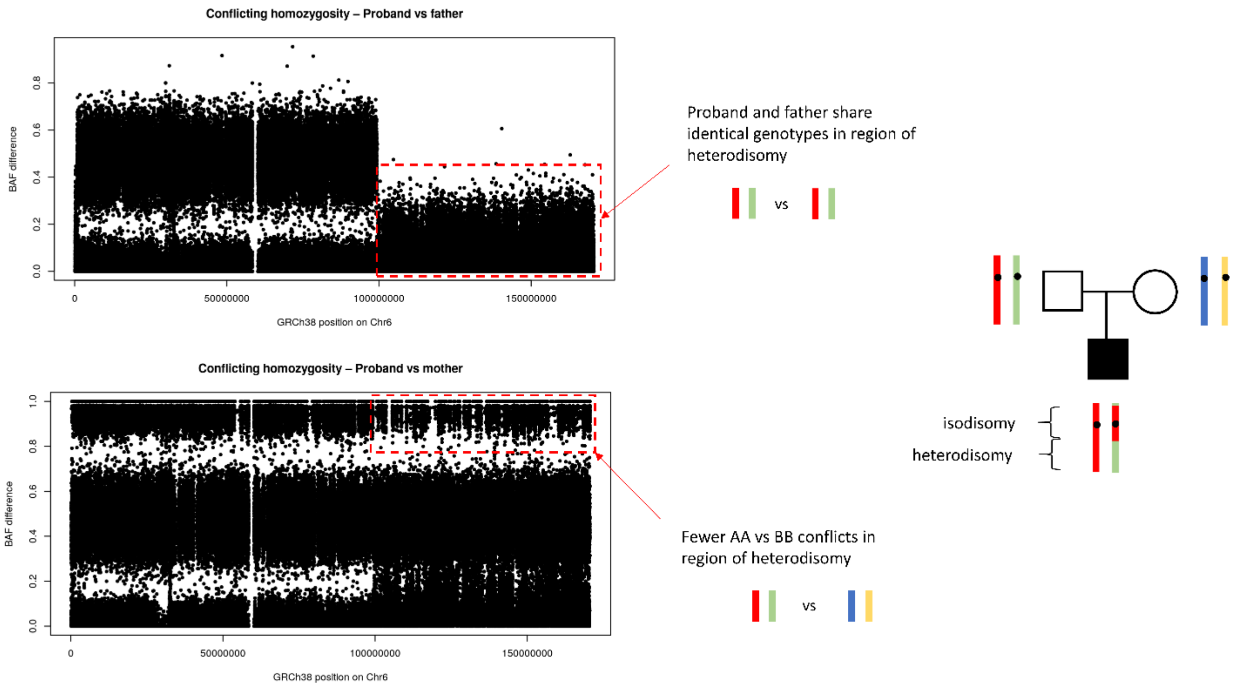

Supplementary Figure 2: Comparative BAF plots highlight the distribution of iso- and heterodisomy regions on chromosome 6. The Y-axis data represents the absolute difference in BAF for the same set of 210,414 SNVs as plotted in Figure S1. In the region of heterodisomy, the proband and the father share both chromosomes in common so genotypes are identical. In contrast, for the proband vs mother comparison, conflicting homozygosity is seen along the entire length of the chromosome.

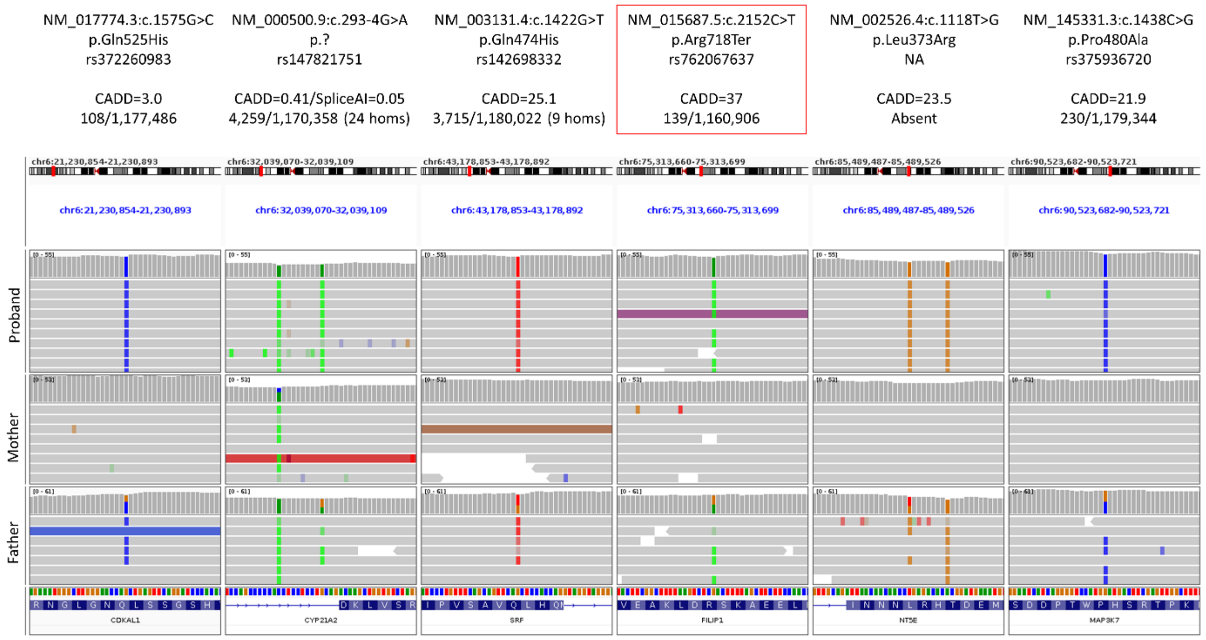

Supplementary Figure 3: Annotation and read-alignments supporting 6 candidate variants in the region of paternal isodisomy on chromosome 6 in Family 1. These variants were prioritised as TIER3 by Genomics England's clinical pipeline due to being potentially protein disrupting and having an allele frequency of <1%. Allele frequency information shown is from the non-Finnish Europeans in gnomAD v4.0.0. The SpliceAI score for the *CYP21A2* variant corresponds to a prediction for an acceptor gain with delta position of 20. The *FILIP1* variant suspected to be responsible for the multiple contractures observed in the proband is highlighted in red and was observed in 38/39 reads.

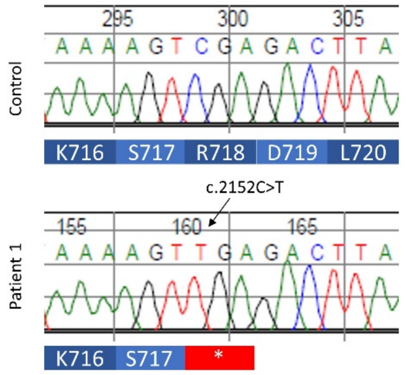

Supplementary Figure 4: Sanger validation data for Family 1. The homozygous NM_015687.5(*FILIP1*):c.2152C>T variant is seen in the patient but not in a control DNA sample. This variant introduces a UGA stop codon in place of the CGA arginine codon, i.e. p.(Arg718Ter).

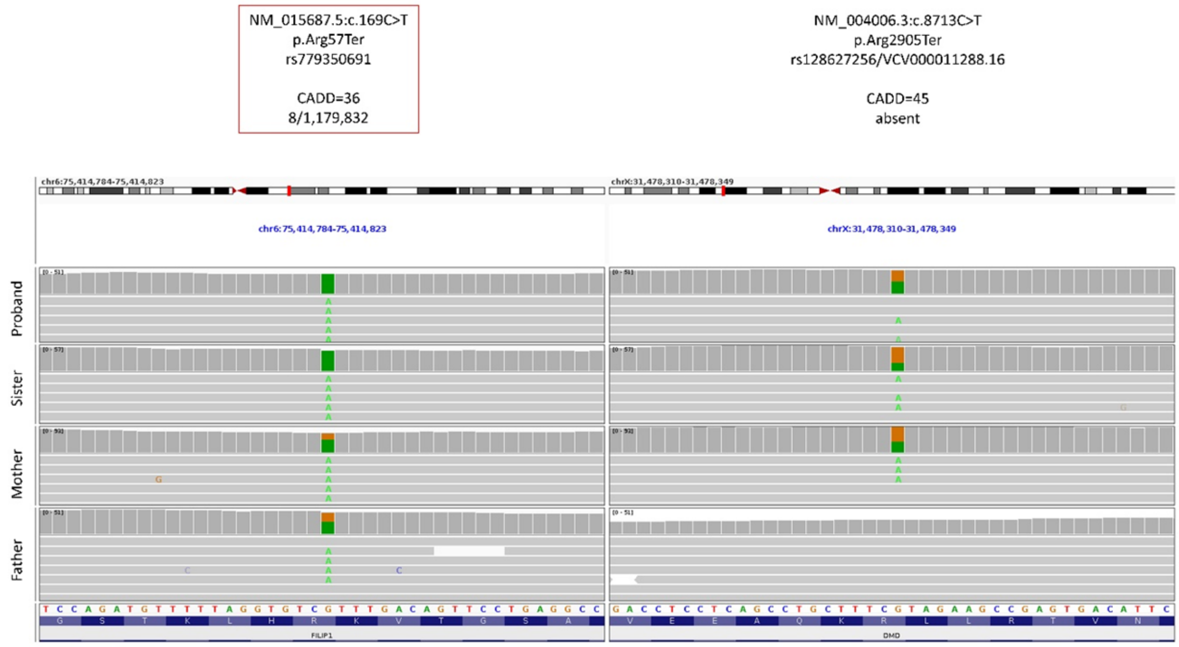

Supplementary Figure 5: Annotation and read-alignments supporting *FILIP1* and *DMD* variants in Family 2. The variant suspected to be primarily responsible for the multiple contractures observed in the proband is highlighted in red although we cannot rule out that the known pathogenic variant in *DMD* is also contributing in some way. Allele frequency information shown is from non-Finnish Europeans in gnomAD v4.0.0.

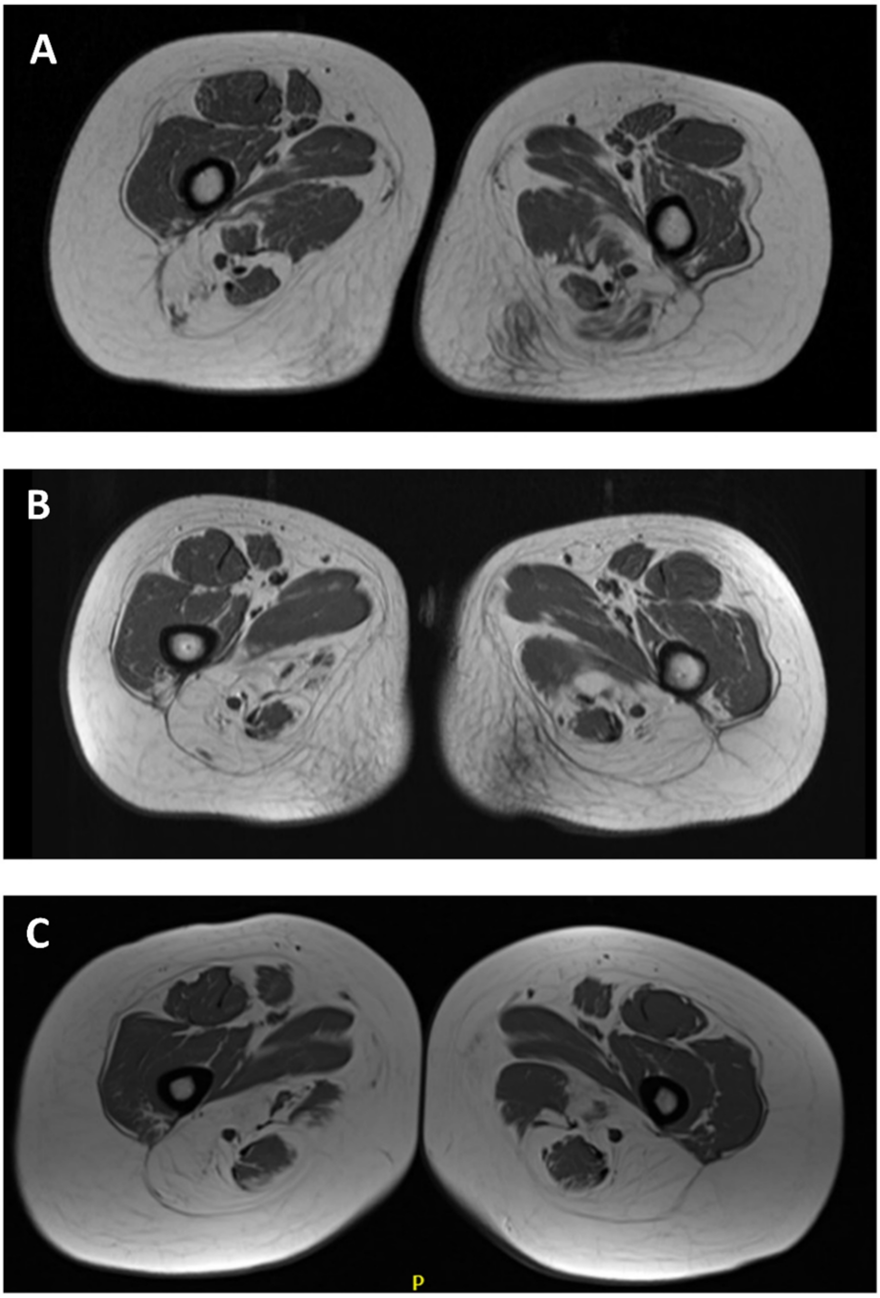

Supplementary Figure 6: MRI images available for Family 2 shows muscle atrophy and variable levels of fatty infiltration. A) Bilateral symmetrical fatty atrophy of the gluteus maximus in younger sister at 11 years. B) Almost complete fatty replacement of the gluteus maximus muscle and quadratus femoris muscle in the older sister at 13 years. The gracilis muscle bilaterally is almost completely replaced by fat. C) Repeat MRI for the older sister at 22 years demonstrates a proximal pattern of muscle atrophy involving the thigh musculature. On the right there is widespread fat atrophy affecting all 3 compartments of the thigh. On the left there is relative sparing of the adductor but involvement of the quadriceps and hamstring compartments is again shown. There is sparing of the musculature of the lower leg and overall no evidence for inflammatory changes.

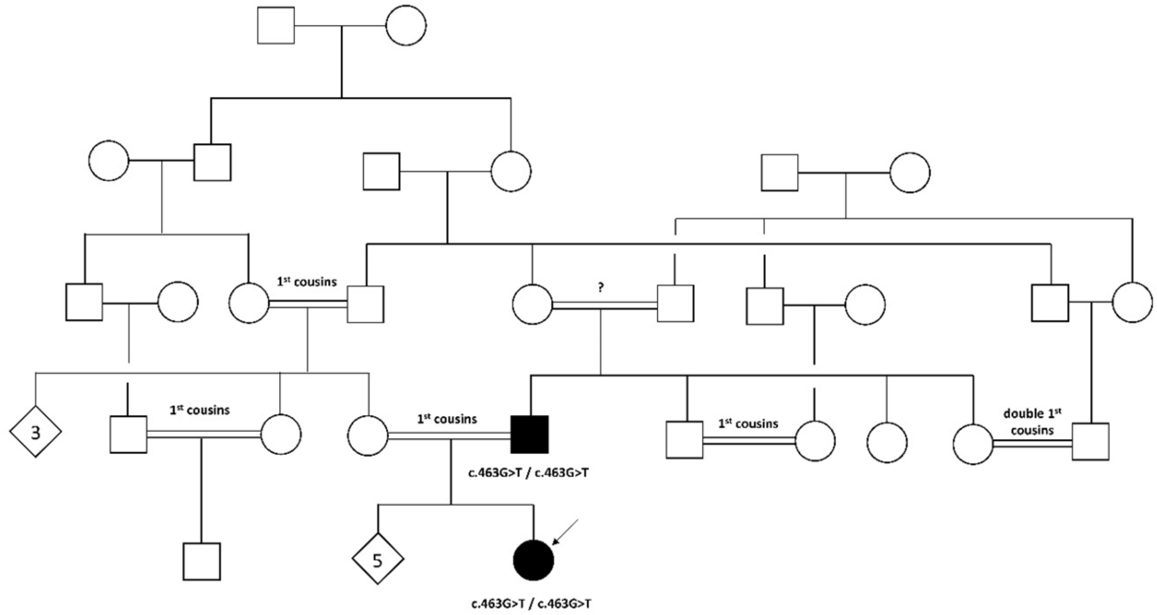

Supplementary Figure 7: Pedigree diagram for multiply consanguineous Pakistani kindred ascertained from the NHS Genome Medicine Service (Family 3). Shading indicates individuals with multiple contractures of joints/fingers. High-confidence genotypes for an ultra-rare NM_015687.5:c.463G>T p.(Glu155Ter) variant in *FILIP1* are indicated based on genome sequencing. Genotypes for unaffected family members are unknown, although the proband's mother and paternal grandparents are obligate carriers. Disease recurrence is possible in other branches where additional 1st cousin marriages have occurred. Although we note that the same variant was reported for Family 1 in Schnabel *et al*[9], we were unable to directly link these families.

References

1. Lander, E.S., and Botstein, D. (1987). Homozygosity mapping: a way to map human recessive traits with the DNA of inbred children. Science 236, 1567-1570.
2. Wakeling, M.N., Laver, T.W., Wright, C.F., De Franco, E., Stals, K.L., Patch, A.M., Hattersley, A.T., Flanagan, S.E., Ellard, S., and Study, D.D.D. (2019). Homozygosity mapping provides supporting evidence of pathogenicity in recessive Mendelian disease. Genet Med 21, 982-986.
3. Yamazawa, K., Ogata, T., and Ferguson-Smith, A.C. (2010). Uniparental disomy and human disease: an overview. Am J Med Genet C Semin Med Genet 154C, 329-334.
4. McQuillan, R., Leutenegger, A.L., Abdel-Rahman, R., Franklin, C.S., Pericic, M., Barac-Lauc, L., Smolej-Narancic, N., Janicijevic, B., Polasek, O., Tenesa, A., et al. (2008). Runs of homozygosity in European populations. Am J Hum Genet 83, 359-372.

5. Rimmer, A., Phan, H., Mathieson, I., Iqbal, Z., Twigg, S.R.F., Consortium, W.G.S., Wilkie, A.O.M., McVean, G., and Lunter, G. (2014). Integrating mapping-, assembly- and haplotype-based approaches for calling variants in clinical sequencing applications. Nat Genet 46, 912-918.
6. Smedley, D., Smith, K.R., Martin, A., Thomas, E.A., McDonagh, E.M., Cipriani, V., Ellingford, J.M., Arno, G., Tucci, A., Vandrovcova, J., et al. (2021). 100,000 Genomes Pilot on Rare-Disease Diagnosis in Health Care - Preliminary Report. N Engl J Med 385, 1868-1880.
7. Roos, A., van der Ven, P.F.M., Alrohaif, H., Kolbel, H., Heil, L., Della Marina, A., Weis, J., Assent, M., Beck-Wodl, S., Barresi, R., et al. (2023). Bi-allelic variants of FILIP1 cause congenital myopathy, dysmorphism and neurological defects. Brain 146, 4200-4216.
8. Al-Kasbi, G., Al-Murshedi, F., Al-Kindi, A., Al-Hashimi, N., Al-Thihli, K., Al-Saegh, A., Al-Futaisi, A., Al-Mamari, W., Al-Asmi, A., Bruwer, Z., et al. (2022). The diagnostic yield, candidate genes, and pitfalls for a genetic study of intellectual disability in 118 middle eastern families. Sci Rep 12, 18862.
9. Schnabel, F., Schuler, E., Al-Maawali, A., Chaurasia, A., Syrbe, S., Al-Kindi, A., Bhavani, G.S., Shukla, A., Altmuller, J., Nurnberg, P., et al. (2023). Homozygous loss-of-function variants in FILIP1 cause autosomal recessive arthrogryposis multiplex congenita with microcephaly. Hum Genet 142, 543-552.
